# Heat and emergency room admissions in the Netherlands

**DOI:** 10.1186/s12889-017-5021-1

**Published:** 2018-01-05

**Authors:** Joris Adriaan Frank van Loenhout, Tefera Darge Delbiso, Anna Kiriliouk, Jose Manuel Rodriguez-Llanes, Johan Segers, Debarati Guha-Sapir

**Affiliations:** 1 Centre for Research on the Epidemiology of Disasters (CRED), Institute of Health and Society, Université catholique de Louvain, Clos Chapelle-aux-Champs 30, 1200 Woluwé-Saint-Lambert, Brussels Belgium; 2 Institute of Statistics, Biostatistics and Actuarial Sciences (ISBA), Université catholique de Louvain, Louvain-la-Neuve, Belgium; 3 European Commission, Joint Research Centre, Directorate for Sustainable Resources, Ispra, Italy

**Keywords:** Heat, Heatwave, Climate, Hospitalization, Respiratory diseases, Circulatory diseases

## Abstract

**Background:**

Due to a global warming-related increase in heatwaves, it is important to obtain detailed understanding of the relationship between heat and health. We assessed the relationship between heat and urgent emergency room admissions in the Netherlands.

**Methods:**

We collected daily maximum temperature and relative humidity data over the period 2002–2007. Daily urgent emergency room admissions were divided by sex, age group and disease category. We used distributed lag non-linear Poisson models, estimating temperature-admission associations. We estimated the relative risk (RR) for urgent hospital admissions for a range of temperatures compared to a baseline temperature of 21 °C. In addition, we compared the impact of three different temperature scenarios on admissions using the RR.

**Results:**

There is a positive relationship between increasing temperatures above 21 °C and the RR for urgent emergency room admissions for the disease categories ‘Potential heat-related diseases’ and ‘Respiratory diseases’. This relationship is strongest in the 85+ group. The RRs are strongest for lag 0. For admissions for ‘circulatory diseases’, there is only a small significant increase of RRs within the 85+ age group for moderate heat, but not for extreme heat. The RRs for a one-day event with extreme heat are comparable to the RRs for multiple-day events with moderate heat.

**Conclusions:**

Hospitals should adjust the capacity of their emergency departments on warm days, and the days immediately thereafter. The elderly in particular should be targeted through prevention programmes to reduce harmful effects of heat. The fact that this increase in admissions already occurs in temperatures above 21 °C is different from previous findings in warmer countries. Given the similar impact of three consecutive days of moderate heat and one day of extreme heat on admissions, criteria for activation of national heatwave plans need adjustments based on different temperature scenarios.

**Electronic supplementary material:**

The online version of this article (10.1186/s12889-017-5021-1) contains supplementary material, which is available to authorized users.

## Background

Over the last 35 years, there has been a global increase in temperatures [1]. One consequence of this global warming is an increase in the frequency and intensity of heatwaves in Europe [2]. Therefore, it is becoming ever more important to gain insights into the effects of extreme heat on health, to guide evidence-based effective planning of preventive and response plans. A number of studies have assessed the relationship between extreme heat and mortality in Europe. Baccini et al. found that high ambient temperatures were responsible for excess deaths in 14 European cities [3]. An increase in mortality has been shown in the population over 50 in Rome and Stockholm for the period 2000–2008 [4]. In a multi-country study, Gasparrini et al. found a higher mortality during cold periods than during warm ones, and a higher contribution from milder non-optimal weather than from extreme temperatures [5]. Yet, extremely high temperatures contributed to an increased mortality. Fewer studies have looked at the impact of extreme heat on morbidity in European countries. Studies using syndromic surveillance showed an increase in the prevalence of certain health conditions during a heatwave in 2013 in England (e.g. heatstroke, sunstroke) [6], and a significant increase in the number of elderly patients [7]. Michelozzi et al. reported an increase in the number of respiratory admissions in 12 European cities, in contrast to the number of cardiovascular and cerebrovascular admissions, which tended to be negatively related to heat or not related at all [8]. Similar results were found by Mastrangelo et al., who observed an increase in respiratory and heat diseases during heatwaves, but not of circulatory diseases [9]. A time series analysis carried out on data from London showed an increase in emergency room admissions for respiratory and renal diseases in children under 5, and for respiratory diseases in the 75+ age group, although a general significant increase was not found [10].

In the Netherlands, winters are mild and summers are relatively cool, due to its maritime climate. These marine influences are slightly less prominent inland, with little variation within the country. The Netherlands has a National Heatwave Plan, which features warnings and notifications of adaptation actions for the most vulnerable groups, aiming at reducing the avoidable public health consequences of heatwaves [11]. There is some literature on the topic of heat and health in the Netherlands. A study by Huynen et al. showed a V-shaped relationship between temperature and mortality, with an optimum 24-h average temperature of 16.5 °C for individuals of 65 years or older [12]. For temperatures above the optimum, mortality increased for malignant neoplasms, cardiovascular disease, respiratory diseases, and total mortality. The number of individuals who died due to a very severe heatwave in 2003 in the Netherlands was estimated to be between 1400 and 2200, mostly elderly in nursing homes [13]. The most commonly reported heat-related symptoms among the elderly in a Dutch study were sleep disturbance (62%), fatigue (61%) and breathing discomfort (29%) [14]. A study by van Loenhout et al. found a significant relationship between heat exposure (indoor and outdoor) and self-reported health problems, such as breathing discomfort and sleep disturbance [15].

From our review, no morbidity study has been carried out so far on the relationship between temperature and an objective indicator, such as hospital admissions, in the Netherlands. Therefore, we assessed the relationship between heat and urgent emergency room admissions by certain disease categories, age groups and sex. In addition, we assessed whether a single-day event with extreme heat leads to a higher risk of being admitted than a multiple-day event with moderate heat. The latter distinction can have important public health implications in terms of warning activation to the population at risk. The lessons we will draw from this study will inform health policy in many countries.

## Methods

This study is based on distributed lag non-linear Poisson models, using data from the Netherlands over the period 2002 to 2007. This period was chosen because it contains summers in which extreme heat events occurred, namely 2003 and 2006 [16]. The area of the Netherlands is 41,543 km^2^, and the population size during the period 2002 to 2007 consisted of approximately 16 million inhabitants [17].

### Data collection

Since we were interested in the relationship between heat and urgent emergency room admissions, we only used a data window covering summer months in which heat events may occur in the Netherlands, namely from May 1st until September 30th.

We collected weather data through the Royal Dutch Meteorological Institute (KNMI), from the official measuring station in De Bilt. Since this station is located in the center of the Netherlands, this station is most representative for the country, and its data could be used as a proxy for countrywide temperatures. We used daily maximum temperatures and mean relative humidity. The latter was calculated by averaging 24-hourly values.

Data on urgent emergency room admissions were obtained from Dutch Hospital Data (DHD). DHD was founded with the aim of managing and maintaining data collection files from all Dutch hospitals (*n* = 112 in the year 2002) [18]. Hospitals provided DHD with monthly updates containing medical data, including data on urgent emergency room admissions by ICD-9 code. The data in our study consist of the daily number of admissions over the period 2002–2007 (*n* = 918 days) from all hospitals combined. Admissions are classified as urgent when they cannot be postponed, since treatment or care is required within the following 24 h [18]. Admissions were further divided by sex (male/female), age group (0–14, 15–64, 65–84, 85+ years of age), and disease category. Only a selected number of disease categories were included, based on studies by Semenza et al. and Mastrangelo et al. [9, 19]. The ICD-9 codes and our descriptive names are presented below:

- ICD 276, 584, 992 (Potential heat-related diseases)
- ICD 460–519 (Respiratory diseases)
- ICD 390–459 (Circulatory diseases)
- ICD 820–821 (Fractures of femur)

The groups ‘Respiratory diseases’ and ‘Circulatory diseases’ correspond with the official ICD-9 classification. The group ‘Potential heat-related diseases’ consists of the subgroups ‘Disorder of electrolyte, fluid and acid-base balance’, ‘Acute renal failure’ and ‘Effects of heat and light’. The group ‘Fractures of femur’ was used as a reference group, since our prediction was that there would not be an increase in the number of fractures due to heat.

### Statistical analysis

Zanobetti et al. (2000) introduced the generalized additive distributed lag models by combining generalized additive models (regression models that allow unspecified smooth functions of the covariates) and distributed lag models (models that relate the event to lagged values of a time-dependent predictor variable) [20]. Armstrong (2006) and Gasparrini et al. (2010) generalized this class of models to distributed lag non-linear models (DLNMs), which allow to describe the dependency along the range of the predictor and along the range of the lag space in a non-linear fashion [21, 22]. This is done by choosing a so-called crossbasis, a two-dimensional functional space, describing the relationship in the two dimensions of predictor and lags.

Let Y_t_ denote the number of hospital admissions on day t and let T_t_ denote the maximal temperature on day t. In our model, we used natural cubic splines, as it provides less biased results than penalized or smoothing splines [23]. Moreover, the natural cubic spline is constrained to be linear at the boundaries of the data range, where data are usually sparse.

We used a generalized linear model from the quasi-Poisson family to allow for overdispersion, i.e., the variance can be larger than the mean. Our model includes an intercept, covariates, a smooth function of time to model seasonal variation and two crossbases. The first crossbasis, for maximal daily temperatures, assumes a natural cubic spline with two degrees of freedom in the temperature space and a natural cubic spline with three degrees of freedom in the lag space. The maximum lag was set to 21 days. This crossbasis is denoted by cb_T_ (T_t_,...,T_t − 21_). To remove remaining autocorrelation, we also included lagged hospital admissions in the model. We again used a crossbasis based on two natural cubic splines, with two and three degrees of freedom for the space of hospital admissions and the lag space, respectively. This crossbasis is denoted by cb_Y_ (Y_t − 1_,...,Y_t − 21_).

The smooth function of time was modelled by a natural cubic spline with 25 degrees of freedom, denoted ns (time, 25), and we use the following covariates: the day of the week; an indicator for holiday (Ascension and Pentecost); the calendar year. We also include a natural cubic spline with 7 degrees of freedom of the relative humidity. Finally, we can write down our model as follows:

Log E [Y_t_] = α + covariates + ns (time, 25) + ns (relative humidity, 7) + cb_T_ (T_t_,...,T_t − 21_) + cb_Y_ (Y_t − 1_,...,Y_t − 21_).

The analyses were performed in the statistical software environment R [24], relying on the package dlnm [25].

Allowing for overdispersion, we used the modified Akaike Information Criterion (AIC) [26] to choose the covariates to include in the model and the number of lags for the two crossbases (i.e. the model with the lowest AIC value is the best model). For the natural cubic splines, we considered degrees of freedom between 2 and 30, and chose the ones giving the lowest AIC. After having fitted the model, we assessed its goodness-of-fit by visual inspection of the deviance residuals, which produces a good fit if 95% of the deviance residuals are between −2 and 2 and no big outliers are present.

## Results

### Exploratory analyses

Because preliminary analyses showed similar behavior for both sexes for all disease categories, we performed the analyses for men and women combined. Datasets were created of the four age groups and four disease categories, 16 in total.

We estimated the relative risk (RR) for urgent hospital admissions for a range of temperatures compared to a baseline temperature of 21 °C. This temperature was the average maximum temperature over the study period, and the one around which the minimum morbidity value on average for the age groups and disease categories seemed to lie. We initially tested a lag of up to 21 days, after which we found that the bulk of the heat impact on admissions occurs in the first days. Therefore, a lag of up to four days was taken into account, meaning the RR of admissions up to four days after a warm day. The RR was obtained as the exponentiated regression coefficient of the model, and the 95% confidence intervals were based on the normal approximation.

We compared the impact of three different temperature scenarios on admissions using the RR. This was done for each of the 16 datasets, for the following scenarios: 1) a day with a maximum temperature of 32 °C; 2) a day with a maximum temperature of 28 °C, preceded by another day with a maximum temperature of 28 °C; 3) a day with a maximum temperature of 26 °C, preceded by two other days with a maximum temperature of 26 °C. Although theoretical, these scenarios give an estimation of events that are likely to occur within the Netherlands. From the study data, the proportion of days with temperatures above 26 °C, 28 °C and 32 °C were 19.7%, 12.7% and 2.4%, respectively. Therefore, a three- or two-day event with 26 °C or 28 °C, respectively, is quite likely to occur, while the chances for a multiple-day event of 32 °C are very low. The RR assesses the impact on the last day of the series for each scenario. Effect modification by age was tested using Cochran’s Q-test.

### Temperature data

**Fig. 1:**
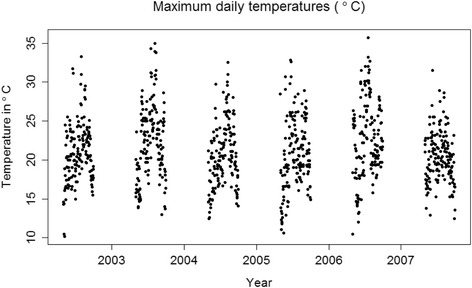
Daily maximum temperatures from May 1st to September 30th between 2002 and 2007 in the Netherlands

Daily maximum temperatures in the Netherlands for May 1st to September 30th over the period 2002–2007 are presented in Fig. 1. Every year, temperatures reached values above 30 °C for at least one or several days. Temperatures above 35 °C were very uncommon within the study period.

### Urgent emergency room admissions

**Fig. 2:**
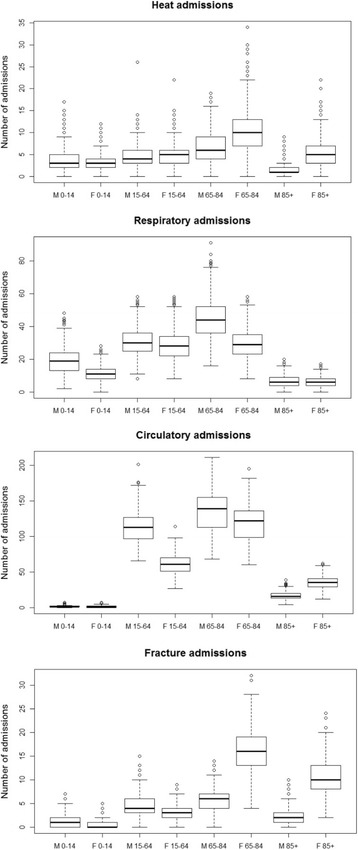
Numbers of daily urgent hospital admissions per disease category, sex (M = Male, F=Female) and age group (0–14, 15–64, 65–84 and 85+ year olds, respectively) from May 1st to September 30th between 2002 and 2007 in the Netherlands

Figure 2 shows boxplots of daily urgent hospital admissions per disease category, sex and age group, for May 1st to September 30th over the study period 2002–2007. Respiratory and especially circulatory disease admissions are numerous, whereas potential heat-related and fracture of femur admissions are sparse, especially for men.

### Heat and urgent emergency room admissions

The deviance residuals of most datasets do not show any remaining pattern. Some outliers are present, mainly for the datasets representing ‘Circulatory diseases’ and ‘Fractures of femur’ of the age group 0–14 (Additional file 1 Annex A).

**Fig. 3:**
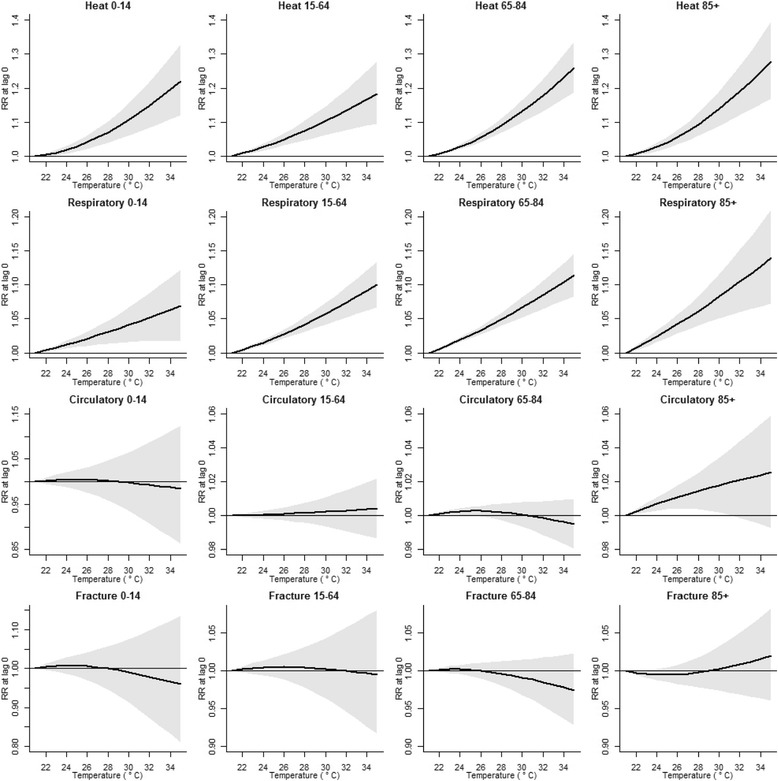
The relative risk (RR) for urgent hospital admissions by temperature compared to a reference temperature of 21 °C, specified by age group and disease category

The RRs for lag 0, representing the direct impact of a warm day, are shown for each dataset in Fig. 3. The contributions of lags 1 to 4 are shown in Additional file 2 Annex B. There is a positive relationship between increasing temperatures above 21 °C and the RR for urgent emergency room admissions for the disease categories ‘Potential heat-related diseases’ and ‘Respiratory diseases’. This relationship is present in all age groups, but strongest in the 85+ group, followed by the 65–84 group. The RRs are strongest for lag 0, but also present for lags 1 to 4. For admissions for ‘Circulatory diseases’, there is only a small increased significant RR within the 85+ age group for moderate heat, but not for extreme heat. There is no significant relationship between ‘Fractures of femur’ and temperature for any of the age groups.

### Impact of different temperature scenarios on admissions

The RRs for three temperature scenarios, for each of the 16 datasets, are presented in Table 1. The RRs on a given day for a one-day event with extreme heat are comparable to the RRs for multiple-day events with moderate heat. For example, for heat-related diseases, the RR for the 85+ group is 1.19 for one day of 32 °C, 1.18 for two days of 28 °C and 1.16 for three days of 26 °C. The effect is mostly visible for the disease categories ‘Potential heat-related diseases’ and ‘Respiratory diseases’. For the group ‘Circulatory diseases’, there is only a small effect within the 85+ group, and for the group ‘Fractures of femur’ there is no effect. The last column of Table 1 shows the *p*-values obtained using Cochran’s Q-test for significance of effect modification by age. These values were calculated using the R package metafor [27]. At the 5% significance level, we can conclude that we reject the null hypothesis of homogeneity for respiratory diseases for the second and third heat scenarios only, whereas at the 1% significance level, the null hypothesis of homogeneity is rejected nowhere.

**Table 1:** The relative risk (RR) for urgent emergency room admissions for three different heat scenarios. The RR was obtained as the exponentiated regression coefficient of the model, and the 95% confidence intervals were based on the normal approximation

		Age 0–14	Age 15–64	Age 65–84	Age 85+	
		RR (95% CI)	RR (95% CI)	RR (95% CI)	RR (95% CI)	*p*-value
Potential heat-related diseases	32 °C, one day	1.15 (1.08 to 1.22)	1.13 (1.08 to 1.20)	1.18 (1.13 to 1.23)	1.19 (1.12 to 1.26)	0.63
	28 °C, two days	1.14 (1.08 to 1.20)	1.14 (1.09 to 1.20)	1.18 (1.13 to 1.22)	1.18 (1.12 to 1.25)	0.64
	26 °C, three days	1.12 (1.07 to 1.17)	1.13 (1.08 to 1.18)	1.15 (1.12 to 1.19)	1.16 (1.10 to 1.22)	0.69
Respiratory diseases	32 °C, one day	1.05 (1.02 to 1.09)	1.07 (1.05 to 1.10)	1.08 (1.06 to 1.11)	1.10 (1.06 to 1.15)	0.22
	28 °C, two days	1.05 (1.02 to 1.09)	1.08 (1.06 to 1.10)	1.09 (1.08 to 1.11)	1.12 (1.08 to 1.16)	0.04
	26 °C, three days	1.05 (1.02 to 1.07)	1.07 (1.06 to 1.09)	1.09 (1.07 to 1.10)	1.11 (1.07 to 1.15)	0.02
Circulatory diseases	32 °C, one day	0.99 (0.91 to 1.09)	1.00 (0.99 to 1.02)	1.00 (0.99 to 1.01)	1.02 (1.00 to 1.04)	0.98
	28 °C, two days	1.00 (0.93 to 1.09)	1.00 (0.99 to 1.01)	1.00 (0.99 to 1.01)	1.03 (1.01 to 1.05)	0.97
	26 °C, three days	1.01 (0.94 to 1.08)	1.00 (0.99 to 1.01)	1.00 (1.00 to 1.01)	1.03 (1.01 to 1.04)	0.97
Fractures of femur	32 °C, one day	0.98 (0.87 to 1.10)	1.00 (0.95 to 1.06)	0.99 (0.95 to 1.02)	1.01 (0.97 to 1.05)	0.98
	28 °C, two days	1.00 (0.90 to 1.11)	1.01 (0.96 to 1.06)	0.99 (0.96 to 1.02)	1.00 (0.96 to 1.03)	0.99
	26 °C, three days	1.01 (0.93 to 1.11)	1.01 (0.97 to 1.05)	1.00 (0.97 to 1.02)	0.99 (0.96 to 1.02)	0.98

*RR* Relative risk, *CI* Confidence interval

Heat scenarios are compared to a baseline temperature of 21 °C, specified by age group and disease category. The RR represents the impact on a single day, i.e. the last day of the temperature scenario. The last column contains *p*-values for Cochran’s Q-test

## Discussion

The most striking finding in our study was that the impact of a single day with extreme heat is comparable to the impact of several days with moderate heat. Heat effects are thus much more profound as they might act in various combinations, producing a much larger attributable health burden than otherwise expected. In addition, our study shows that an increase in temperature during the summer months is associated with an immediate increase in urgent emergency room admissions. This effect is visible for the disease categories ‘Potential heat-related diseases’ and ‘Respiratory diseases’, but almost absent for ‘Circulatory diseases’. All age groups are affected by heat, but the risk of being admitted is highest among the elderly (persons aged 85+).

Our results show that not only single days of extreme heat lead to an increase in admissions, but that the risk of being admitted after a multiple-day event with moderate heat is almost the same. A study by Levy et al. found that a single day has only a limited effect on emergency room admissions, but the effect becomes cumulative after continued hot weather [28]. A study among children in Brisbane, Australia, also showed an increase in hospital admissions by duration of the heatwave [29]. In contrast, a recent meta-analysis showed that for the impact of heatwaves on mortality, intensity of a heatwave is more important than duration [30]. Although national adaptation strategies often put emphasis on short periods with extreme heat, results from our study and Levy et al. indicate that a longer lasting period with moderate heat can be equally dangerous. This should be taken into consideration by policy makers, e.g. in establishing criteria for activation of national heatwave plans.

The risk of being admitted was highest in our study for the disease category ‘Potential heat-related diseases’. A temperature of 30 °C, which is a common occurrence in a typical Dutch summer, as shown in Fig. 1, led to RRs for the same day of more than 1.1, or above 10% increase in admissions (Fig. 3). If the days following a hot day are taken into consideration as well, this effect is even higher (Additional file 2 Annex B). This is in accordance with studies that showed an increase in emergency room admissions for heat-related illnesses on days with high daily maximum temperatures, especially among the elderly [8, 9, 31]. In addition, the relationship between heat and an increase in admissions for ‘Respiratory diseases’, the second most affected group in our study, has been shown in other studies as well [9, 32, 33]. We found no significant relationship between heat and admissions for ‘Circulatory diseases’ for any of the age groups, except for a small effect in the age group 85+ for moderate heat. These results are confirmed by previous studies [9, 34]. A recent meta-analysis found a significantly increased risk for cardiovascular hospital admissions during heatwaves (2 days or more of extreme high temperature), but not during heat exposure [35]. It has been suggested that people die rapidly from circulatory diseases due to heat, before they are admitted [36]. However, studies found significant increases for congestive heart failure admissions in summer months [37], a significant relationship between high temperatures and admissions for acute myocardial infarctions [38], and a marked increase in ischemic heart disease during a severe heatwave [39]. One possible explanation for these differences is that there is an increase in admissions for certain diseases due to heat, but this trend is diluted by the complete group of circulatory admissions. A follow-up study should focus on the relationship between heat and individual diagnoses.

For all datasets in our study, the RR was highest at lag 0. This immediate effect of heat has been confirmed by previous studies [32, 40]. This finding has important implications for hospitals, as they should be able to immediately increase the capacity, in terms of beds and medical personnel, of their emergency departments on warm days.

Our study did not identify a different risk for being admitted between males and females. In terms of age, the highest risks for all disease categories were found in the highest age groups, individuals aged 85 or higher, followed by individuals aged 65 to 84. It has been shown in numerous studies that the elderly, in particular individuals aged 75 or higher, are a risk group for harmful effects due to heat, including hospital admissions [8, 10, 41–45]. A possible biological explanation for this is that their threshold for the development of renal failure is lower and their thirst is often impaired, both of which can be aggravated by commonly used medications among this group [46]. Therefore, it is important to target elderly and their caregivers in activities aimed at reducing the harmful effects of heat, such as national heatwave plans [47]. For children aged 0–14, significant relationships were found for the disease categories ‘Potential heat-related diseases’ and ‘Respiratory diseases’. This last finding was in line with other studies that have shown an increase in respiratory admissions due to heat for this age group [48], in particular for childhood asthma [49]. Previous literature has also pointed out that the effects of heatwaves are mostly seen in the subset of children aged 0–4, who have an increased risk for emergency department visits [50]. A stronger effect for this subgroup might be hidden among the larger age group of 0–14 in our study.

Although we used data from the Netherlands, the patterns from our study may be extrapolated to other European settings, and beyond. For example, a study by Baccini et al. on temperature and mortality shows a relationship between these variables, with a similar V-shaped pattern in different European cities, but with different change points [51]. A study from Sydney, Australia, also showed similar findings when comparing a three-day event with severe heat to a one-day event of extreme heat, although the maximum temperatures reached in this setting were much higher than in the Netherlands [52]. Acclimatization and personal susceptibility were indicated as possible explanations for the differences between cities [51]. In addition, local early warning and adaptation measures in place could account for part of this variation. A similar trend can be expected in our study: the direction of the RRs will most likely be the same in different countries, although the temperature scale and dose-response relationship typically differ [4, 51, 53].

This study has some limitations. We could not include air pollution as a covariate in our study, since our data were on a national level. Air pollution can vary greatly among different regions, and in particular between urban and rural areas. For the same reason, it was not possible to make a comparison between temperature and the number of admissions on a regional scale. Our data was divided in four broad age groups. Therefore, we could not assess the impact of heat on admissions of individuals of a particular age, e.g. very small children.

## Conclusions

Individuals aged 85 or higher had the highest RRs of being admitted during warm days, followed by individuals aged 65 to 84, which reconfirms previous studies. The elderly should be targeted in activities to reduce harmful effects of heat. An increase in temperature during the summer months is associated with an immediate increase in urgent emergency room admissions for the disease categories ‘Potential heat-related diseases’ and ‘Respiratory diseases’. This increase in admissions is already found in temperatures above 21 °C, which is remarkably lower compared to previous studies in warmer countries. The operational implications are that hospitals should adjust the capacity of their emergency departments during warm days, especially in densely populated areas. The impact on morbidity of sustained moderate heat should not be underestimated by public health stakeholders, as their consequences can be as important as an intense heat spike. This should be taken into consideration by policy makers, e.g. in establishing criteria for activation of national heatwave plans.

## Additional files

Additional file 1: Annex A. Deviance residuals for each of the 16 datasets by age group and disease category. (DOCX 428 kb)

Additional file 2: Annex B. The relative risk (RR) for urgent emergency room admissions by temperature at lags 0, 1, 2, 3 and 4 compared to a reference temperature of 21 °C, specified by age group and disease category. (DOCX 310 kb)

## Abbreviations

AIC: Akaike Information Criterion
DHD: Dutch Hospital Data
DLNM: Distributed lag non-linear model
ICD: International Classification of Diseases
KNMI: Royal Dutch Meteorological Institute
RR: Relative risk
