# Supplementary material for: Heat and emergency room admissions in the Netherlands
Source: BMC Public Health. 2018 Jan 5;18:108. doi: 10.1186/s12889-017-5021-1 (PMC5756417; doi:10.1186/s12889-017-5021-1)

Additional file 1 Annex A. Deviance residuals for each of the 16 datasets by age group and disease category.


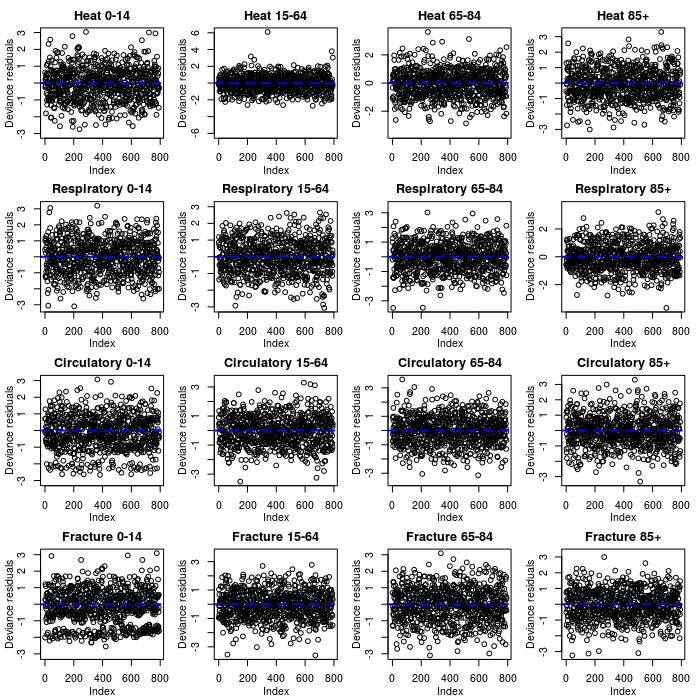

Supplement: Supplementary file 1 — Annex A. Deviance residuals for each of the 16 datasets by age group and disease category. (DOCX 428 kb) [file 12889_2017_5021_MOESM1_ESM.docx]
