# Supplementary material for: Heat and emergency room admissions in the Netherlands
Source: BMC Public Health. 2018 Jan 5;18:108. doi: 10.1186/s12889-017-5021-1 (PMC5756417; doi:10.1186/s12889-017-5021-1)

Additional file 2 Annex B. The relative risk (RR) for urgent emergency room admissions by temperature at lags 0, 1, 2, 3 and 4 compared to a reference temperature of 21 °C, specified by age group and disease category.


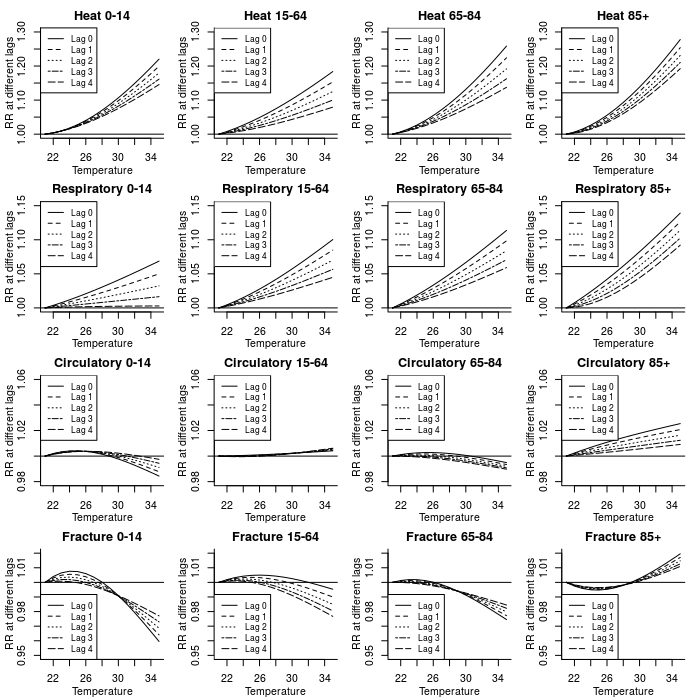

Supplement: Supplementary file 2 — Annex B. The relative risk (RR) for urgent emergency room admissions by temperature at lags 0, 1, 2, 3 and 4 compared to a reference temperature of 21 °C, specified by age group and disease category. (DOCX 310 kb) [file 12889_2017_5021_MOESM2_ESM.docx]
